# A genetic characterization of Korean waxy maize (*Zea mays* L.) landraces having flowering time variation by RNA sequencing

**DOI:** 10.1038/s41598-019-56645-y

**Published:** 2019-12-27

**Authors:** Gibum Yi, Hosub Shin, Seung Hwa Yu, Jeong Eun Park, Taegu Kang, Jin Hoe Huh

**Affiliations:** Department of Plant Science and Plant Genomics and Breeding Institute, Seoul National University, Seoul, 08826 Korea

**Keywords:** Gene expression, Plant breeding

## Abstract

Maize is the second-most produced crop in the Korean peninsula and has been continuously cultivated since the middle of the 16th century, when it was originally introduced from China. Even with this extensive cultivation history, the diversity and properties of Korean landraces have not been investigated at the nucleotide sequence level. We collected 12 landraces with various flowering times and performed RNA-seq in the early vegetative stage. The transcriptomes of 12 Korean landraces have been analyzed for their genetic variations in coding sequence and genetic relationships to other maize germplasm. The Korean landraces showed specific genetic characteristics and were closely related to a Chinese inbred line. Flowering-time related gene profiles pointed to multiple causes for the variation of flowering time within Korean landraces; the profiles revealed significant positive and negative correlations among genes, allowing us to infer possible mechanisms for flowering time variation in maize. Our results demonstrate the value of transcriptome-based genetic and gene expression profiles for information on possible breeding resources, which is particularly needed in Korean waxy landraces.

## Introduction

Maize arrived in Korea from China, as has been documented in several old texts, including the Shilok, an official government document from the Joseon Dynasty (1392–1897; http://sillok.history.go.kr). Maize has been cultivated throughout the Korean peninsula since the middle of the 16th century^1^. Over 3,000 landraces collected throughout South Korea are deposited in the National Agrodiversity Center (Republic of Korea; www.genebank.rda.go.kr). These landraces are characterized phenotypically. However, the origin, genetic diversity, and relationship to other well-known maize lines of Korean landraces have not been investigated at the DNA sequence level. A limited number of studies have used Korean maize landraces^1^. Moreover, Korean landraces have not been included in any of the large-scale population studies so far, such as the third-generation maize haplotype map (HapMap3)^2^.

Maize is a highly diverse crop. Even taking into account the large-scale population studies on Mexican and European landraces or the HapMap3 populations, most maize landraces remain largely uncharacterized^2,3^. Better understanding the characteristics of landraces would be beneficial for addressing the challenges of a changing climate and environment through genetic diversity. Recently, 83 million variant sites have been called from 1,218 lines of maize^2^. Among 55 million genome wide SNPs in 103 lines, 21% were found to be associated with a genic region in maize^4^. Coding sequences are well conserved, but contain sufficient polymorphism to determine genetic relationships among maize lines^5^. In addition, RNA sequencing can be used to detect polymorphisms in transcribed sequences and to determine transcript profiles during specific developmental stages^6,7^.

Flowering time is important for successful environmental adaptation, varies greatly among landraces, and can be affected by geographic factors such as latitude and altitude. Flowering time is an important trait not only for environmental adaptation but also for yield in maize. The genes and QTLs for flowering time regulation have been revealed with population genetics approaches, verifying that flowering time in maize has adapted to different environments^3,8^. Thus, flowering time is an ideal trait for characterizing the genetic diversity of local landraces.

This study was performed with two main objectives: first, to place Korean landraces into the HapMap2 population^4^ to assess their genetic characteristics using SNPs from RNA-seq. Second, to use transcript profiling to assess the variation in expression of flowering-time associated genes across landraces with varying flowering times.

## Materials and Methods

### Plant materials and phenotyping

Twelve Korean landraces were selected based on a large variation on their flowering time, and B73 was used as a control (Table 1). The selected landraces were all waxy maize, which is the preferred texture type and likely to be conserved genetically in South Korea. Aboveground tissues at the V2 stage, when the second but not the third leaf was liguled, were sampled. Maize plants used for RNA-seq were grown in a growth room at 24 °C under a 16-h day. Sampling was done at 10–11 a.m. in all samples to minimize gene expression differences caused by circadian rhythm. Three biological replicates were sampled for each landrace. Flowering time was measured in a Seoul National University farm field in Suwon, Korea (37°16ʹN 126° 59ʹE) in 2018. Flowering time was determined by the emergence of pollen shedding anther or silks. The average number of days after planting to flowering was measured for 10 plants. Plant height was measured from the soil line to the top of the plant, and leaf number and number of tillers were counted at the time of anthesis. The means ± standard deviations were calculated from these 10 individuals.

**Table 1:** Phenotypes of accessions used in this study.

IT No.	Name	Anthesis (DAS)	Silking (DAS)	Stalk height (cm)	No. of leaves (ea)	No. of tillers (ea)
IT124603	B73	76.3 ± 1.0	77.7 ± 1.4	209.1 ± 3.0	15.0 ± 3.0	0.3 ± 0.5
IT208593	Seocheon Chal	57.9 ± 1.6	55.9 ± 2.0	144.0 ± 5.4	9.6 ± 0.5	0.7 ± 0.7
IT262719	Pyeongchang Chal-14	60.3 ± 1.7	59.4 ± 2.3	187.5 ± 13.2	10.5 ± 0.7	1.5 ± 1.0
IT229632	Okcheon Chal-1	64.0 ± 2.0	64.4 ± 2.5	169.3 ± 11.4	11.1 ± 0.7	1.4 ± 1.0
IT195284	Goseong Chal	65.7 ± 3.3	67.0 ± 3.0	188.3 ± 8.0	11.3 ± 1.2	1.5 ± 1.0
IT195253	Wonju Chal	70.1 ± 1.3	71.0 ± 1.0	254.5 ± 9.8	13.6 ± 0.7	2.0 ± 0.7
IT262740	Hoengseong Chal-9	70.4 ± 0.5	70.6 ± 0.7	216.3 ± 10.2	14.0 ± 1.1	1.1 ± 0.6
IT026475	Jeongseon Chal-1	70.5 ± 1.0	74.8 ± 2.0	235.0 ± 4.1	13.6 ± 0.5	1.0 ± 0.8
IT026484	Inje Chal-1	73.8 ± 1.8	76.7 ± 1.2	224.2 ± 10.2	15.4 ± 1.0	2.0 ± 0.0
IT229630	Cheongyang Chal	82.3 ± 1.6	84.6 ± 0.9	184.0 ± 3.7	17.0 ± 0.5	2.6 ± 0.7
IT026532	Hoengseong Chal-3	84.0 ± 0.8	89.2 ± 0.4	151.7 ± 6.2	17.3 ± 0.4	1.0 ± 0.8
IT229631	Dangjin Chal	86.0 ± 1.1	88.3 ± 1.0	179.9 ± 4.2	16.4 ± 0.7	2.3 ± 0.7
IT178746	Misang Chal-8	91.1 ± 0.6	97.7 ± 0.7	158.1 ± 5.4	16.6 ± 0.9	3.1 ± 0.6

### RNA-seq analysis

Total RNA was extracted with the Plant RNeasy mini kit (Qiagen, Germany) and DNase treated on column according to the manufacturer’s protocol. A Truseq RNA library was constructed according to the manufacturer’s protocol (Illumina, USA). Raw sequencing reads were generated by the HiSeq. 4000 system (Illumina, USA). Sequence data were deposited NCBI (PRJNA511900). High quality reads were collected using FASTX-Toolkit with –q 30 –p 80 options. Cleaned reads were mapped onto the B73 RefGen_v4 genome (https://www.maizegdb.org) using Tophat^9^ with default parameters. The mapped read counts were calculated by HTseq.^10^. TMM normalization of EdgeR^11^ was performed and FPKM (fragments per kilobase of transcript per million reads) was calculated.

### SNP analysis

For detecting SNPs, cleaned reads were aligned using the STAR program^12^, and the Genome Analysis Toolkit (GATK)^13^ was used for variant calling following the Best Practices workflow for RNAseq data. SNPs with a read depth of less than 5 were eliminated. To determine the relationship between the 12 maize lines from Korea and other maize lines, 113 maize lines from the Maize HapMap2 population were compared. The SNP calling data of 113 HapMap2 individuals were extracted from HapMap3^2,4^. To examine the SNPs from RNA-seq data and whole genome sequence-based SNPs from HapMap2, we called the genotypes of read mapping regions from RNA-seq by GATK HaplotypeCaller with the –GVCF option and only positions overlapping SNPs from RNA-seq and SNPs from HapMap2 were selected.

### Pedigree analysis

To identify the genetic relationships among 126 maize lines, a neighbor joining tree was constructed using MEGA X^14^. Conserved SNP positions among 13 RNA-seq maize lines from this study and 113 maize lines from HapMap2 were used and genetic distances were computed by the p-distance method. A principal component analysis (PCA) of SNPs within 13 RNA-seq maize lines was also performed using the SNPRelate R package^15^. TASSEL 5.0^16^ was used for visualizing SNPs and analyzing SNP genotypes.

### Genome-wide association analysis

Detected SNPs were filtered out when they are located within 100 bp, or their minor allele frequency is lower than 0.05. Genome-wide association for phenotypes was measured with weighted Mixed Linear Model by TASSEL 5.0^16^.

### Expression profiling and correlation analysis

The weighted gene co-expression network analysis (WGCNA) was performed with the WGCNA R package^17^. Genes having an average of FPKM > 0.3 were used for module detection. Twenty-four modules were obtained by the automatic network construction function with default parameters except for the soft power of 8, the minimum module size of 30, and the merge cut height of 0.45. A heatmap was drawn with web-based -omics data analysis program Metaboanalyst (http://metaboanalyst.ca)^18^. Correlation analysis and statistical tests were performed with EXCEL and R software (v 3.2.1).

## Results

### Phenotypes of Korean landraces

To cover a range of genetic variations, 12 landraces were selected based on their flowering time (Table 1). The landraces were categorized as early, moderate, or late flowering (www.genebank.rda.go.kr). They flowered from 57.8 to 91.1 days after sowing (DAS) for anthesis and from 55.9 to 97.7 DAS for silking. In the same field, B73 flowered at 76.3 and 77.7 DAS for anthesis and silking, respectively (Table 1). The anthesis-silking interval was 2 days on average, ranging from 0.2 to 6.6 days. Stalk height (from 144.0 to 254.5 cm), number of leaves (from 9.6 to 17.3), and number of tillers (from 0.7 to 3.1) were measured with the same plants at the time of flowering. The highest correlations were observed between anthesis and silking (Pearson’s *R* = 0.992, p < 0.0001), leaf number and anthesis (Pearson’s *R* = 0.946, p < 0.001), and leaf number and silking (Pearson’s *R* = 0.953, p < 0.001). The total leaf number was generally a good indicator for flowering time: 9.6 leaves on average for the earliest flowering Seocheon Chal and 16.6 leaves on average for the latest flowering Misang Chal-8. However, there were some exceptions. For example, Hoengseong Chal-3 had 0.7 more leaves on average than Misang Chal-8, but it flowered a week earlier. Plant height did not significantly correlate with any other trait. There were positive correlations with low significance between tiller number and anthesis (Pearson’s *R* = 0.625, p < 0.05) and between tiller number and silking (Pearson’s *R* = 0.600, p < 0.05). These correlations are likely because the tiller number was measured at the time of flowering, and thus the late-flowering plants had a longer period of growth to initiate tillers.

### SNP detection in Korean landraces

We obtained a total of 1,648 million reads from 39 samples (13 lines × 3 reps) ranging from 36.1 to 64.7 million reads with 42.3 million reads on average (Supplementary Table 1). B73 showed the highest rate of concordant pairing to the reference B73; however, the rate was only 87.8%, suggesting possible errors in sequencing and mapping. Perhaps more importantly, the B73 (IT124603, Table 1) from the Korean Agrodiversity center is slightly different from the reference B73 (Supplementary Table 1). The Korean landraces had rates of concordant pairing to B73 from 75.4% to 79.4%, demonstrating both their genetic distance to the reference B73 and variations among Korean landraces (Supplementary Table 1).

From the RNA-seq of twelve Korean landraces, 2,604,272 SNPs were detected compared to the reference B73 genome sequence. The reads were mapped to 29,940 genes, representing 75.8% of all annotated genes, and totaled 39.9 Mb in length. Thus, there were 25.3 SNPs/1 kb and 33.7 SNPs/gene.

**Figure 1:**
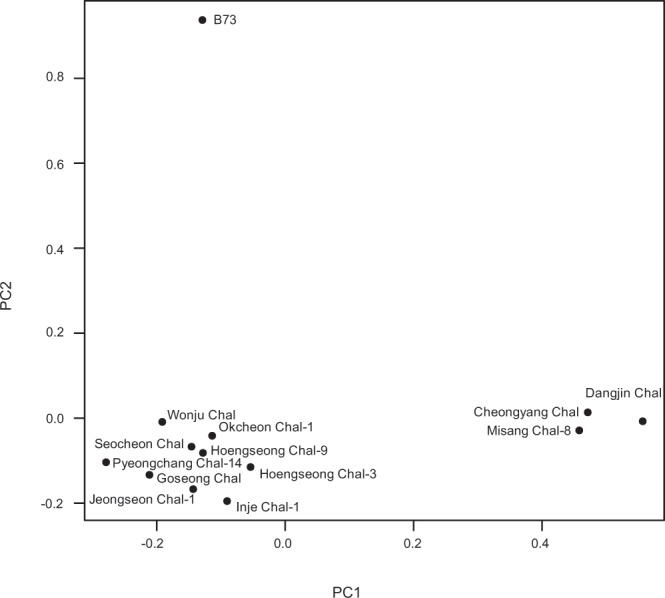
Principal component analysis based on 2,604,272 single nucleotide polymorphisms (SNPs). 13 samples were loaded by their SNP genotypes on the loading plot.

Based on the SNPs, 13 samples were separated into three groups by PCA (Fig. 1). B73 was located alone on the top right of the loading plot, indicating the genetic distance between B73 and Korean landraces. There were two clusters in Korean landraces: one cluster included Dangjin Chal, Cheongyang Chal, Misang Chal-8, while the remaining landraces clustered together (Fig. 1). The three maize lines in the first cluster were all late flowering landraces flowering later than 80 DAS. Furthermore, two of these lines originated close to each other in the Middle West of the Korean peninsula, and the other has an unknown geographic origin (Supplementary Fig. S1). When SNP genotypes were applied to the flowering phenotype, no significant association was detected (Supplementary Fig. S2).

### Korean landrace specific clustering in a phylogenetic tree

**Figure 2:**
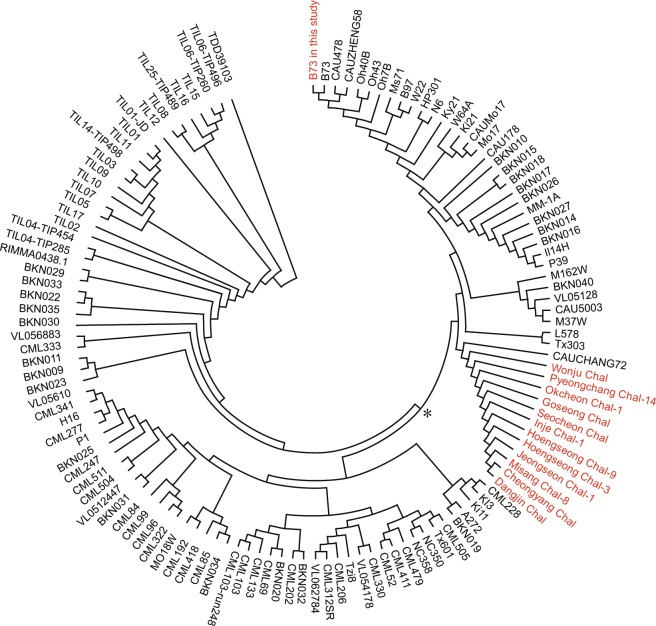
Neighbor-joining tree of 13 maize lines including 12 Korean landraces and 113 HapMap2 lines based on 9,871 single nucleotide polymorphisms (SNPs). The analyses were conducted in MEGA X (Kumar *et al*. 2018). The samples used in this study are colored red. *Indicate the branch dividing tropical and temperate maize.

To place the Korean landraces into the existing phylogenetic tree, we made a haploid map with the 113 HapMap2 lines, which correspond to 2,604,272 SNPs throughout the coding sequence^2,4^. Missing data were trimmed for all samples and 9,871 SNPs were used for drawing a phylogenetic tree. Heterozygosity of the samples was estimated using the 9,871 SNPs. Homozygosity was 93.8% on average and ranged from 89.5% to 99.7%, where Pyeongchang Chal-14 has the lowest and Dangjin Chal has the highest level of homozygosity, respectively. Late flowering landraces tended to have higher homozygosity, which is likely due to isolated seasonal fertilization caused by late flowering. The tree had TDD39103 (*Tripsacum dactiloydes*) as the outmost branch with teosinte (*Z*. *mays*. ssp. *parviglumis* and *mexicana*) as out groups (Fig. 2). The remaining lines were generally separated into two major branches for tropical and temperate lines. This result shows that 9,871 selected SNPs from genic regions are sufficient to separate HapMap2 lines similarly to whole-genome comparison of SNPs. The twelve landraces were clustered together and located in the temperate lowland maize group closest to CAUCHANG72, a Chinese elite inbred, showing the origin of Korean landraces (Fig. 2)^19^. Among Korean landraces, Dangjin Chal, Cheongyang Chal, and Misang Chal-8 were closely located together, confirming their genetic similarities observed in PCA based on 2,604,272 SNPs (Figs. 1 and 2).

### Flowering time determination in Korean landraces

**Figure 3:**
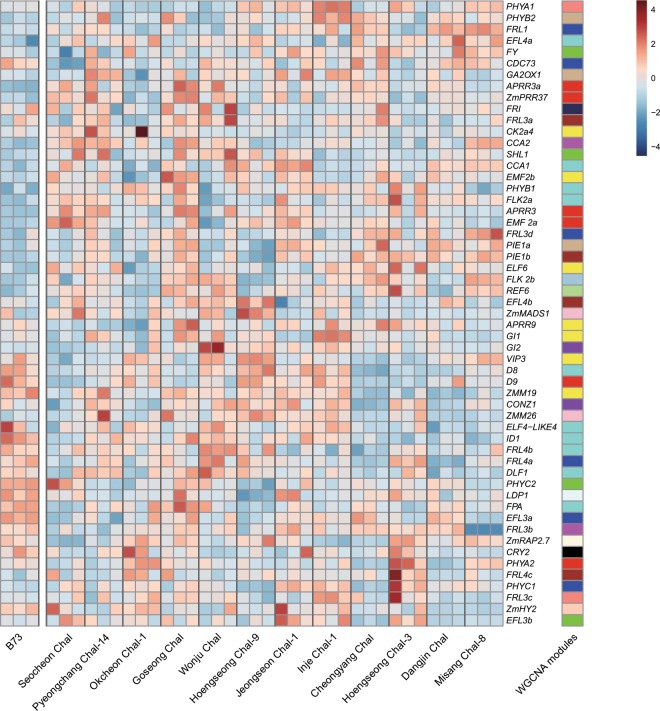
Expression profiles of flowering-time related genes. Auto scaled FPKM values of three plants of B73 and 12 Korean landraces were visualized in colors as indicated. The 12 Korean landraces are arranged by flowering time. The WGCNA modules were color-coded on the right column.

At the V2 stage, temperate maize lines do not switch from the vegetative to the reproductive phase even in very early flowering accessions^20^. We analyzed the expression of genes known to be important for flowering-time determination in maize (Supplementary Table 2). These genes included both cloned genes and candidate flowering time-related genes based on their annotation, and their expression was analyzed for B73 and 12 Korean landraces. We further performed the WGCNA on a whole gene set (average FPKM > 0 .3), and obtained 24 modules of which blue and green modules were positively and negatively correlated with flowering time, respectively (Supplementary Fig. S3). The expression profiles of flowering time genes were diverged within the Korean landraces (Fig. 3). For example, a blue module gene *FRL1* was highly expressed in Dangjin Chal and Misang Chal-8 but not in Hoengseong Chal-3, whereas the other blue module genes *FRL4a* and *PHYC1* were highly expressed only in Hoengseong Chal-3. This divergence suggests that there is not a single causal genotype or genotypic combination behind early flowering or late flowering in the Korean landraces.

Twelve landraces and B73 were grouped based on their flowering-time gene expression profiles by PCA, in which three biological replicates of 13 accessions were placed according to their normalized transcript levels (Supplementary Fig. S4). In most cases, the three biological replicates were located close together, showing their consistency in gene expression. The landraces with larger 95% confidence intervals tended to have lower levels of homozygosity (Supplementary Fig. S4). Moreover, most of the late-flowering landraces were located together on the first quadrant close to the center of the plot, indicating similarities in their flowering-time gene expression (Supplementary Fig. S4). Three of these lines, Dangjin Chal, Cheongyang Chal, and Misang Chal-8 were also closely related according to their SNP genotypes, showing the correlation between genotype and phenotype (Fig. 1).

*ZEA CENTRORADIALIS* (*ZCN8*), an orthologue of Arabidopsis *FT*, expression was detected only in the early-flowering lines Seocheon Chal and Okcheon Chal-1 at a low level (0.3 < FPKM < 1) and not detected in other lines at this developmental stage^21,22^. *ZMM4* (*ZmMADS4*), a MADS-box gene inducing flowering, was not expressed in any samples and is known to increase in expression during the transition to flowering^23^. Floral integrators such as *DLF1* (0.7 < FPKM < 5.0) and *ZmMADS1* (0.4 < FPKM < 5.8) were also downregulated^20,24^. However, autonomous pathway genes such as *ZmFPA*, *LUMINIDEPENDENS PROTEIN1* (*LDP1*), and *INDETERMENATE1* (*ID1*), the first flowering-time gene identified in maize as a transcription factor containing a C2H2 zinc finger domain^25^, were highly expressed. Photoperiod-related genes such as phytochromes, cryptochromes, *ZmHy2*, *conz1*, and circadian clock genes including *ZmPPR3*, *CCA1*, *CCA2*, *GI1*, and *GI2* were also highly expressed. Flowering gene-expression profiles showed that the V2 stage is vegetative in all accessions in this study.

**Figure 4:**
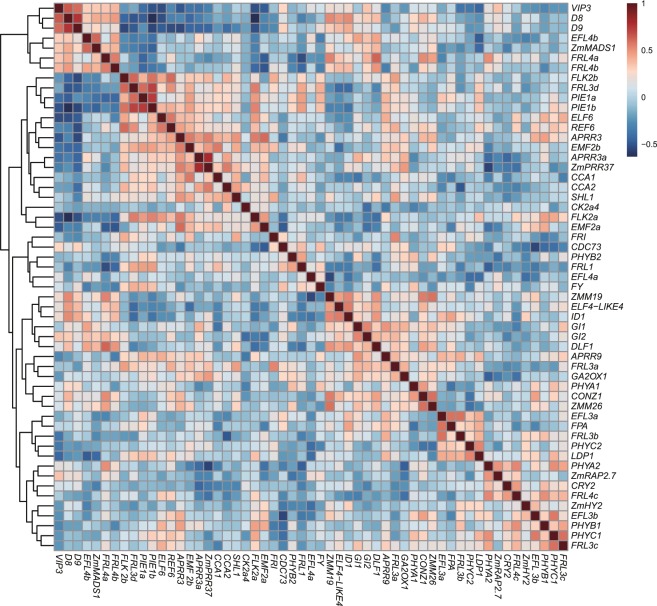
Correlations among flowering-time related genes. The value of Pearson’s *R* is represented by colors as indicated.

Correlations among 55 flowering-time genes (Supplementary Table 2) in the 12 Korean landraces and B73 were analyzed via a heatmap of Pearson’s *R* value (Fig. 4). The expression values in FPKM were transformed by autoscaling to remove the bias between highly- and weakly-expressed genes. The strongest correlations were observed in homologous gene pairs such as *D8* and *D9* (Pearson’s *R* = 0.752, p < 0.001), *APRR3a* and *ZmPRR37* (Pearson’s *R* = 0.794, p < 0.001), *PIE1a* and *PIE1b* (Pearson’s *R* = 0.734, p < 0.001), *EFL6* and *REF6* (Pearson’s *R* = 0.577, p < 0.05), and *GI1* and *GI2* (Pearson’s *R* = 0.534, p < 0.001). These strong correlations between homologous genes suggest they may be co-regulated during flowering-time regulation and may have conserved functions. There were also groups of genes with highly correlated expression levels among the maize lines in this study. For example, *D8*, *D9*, and *VIP3* (Pearson’s *R* > 0.610, p < 0.001) were positively correlated within groups as were *FLK2b*, *FRI-LIKE* (*FRL*) *3d*, *PIE1a*, and *PIE1b* (Pearson’s *R* > 0.406, p < 0.005). It is notable that these two groups of genes were negatively correlated with one another, with Pearson’s *R* ranging from −0.16 to −0.63 and an average value of −0.43. *ZMM19*, *ELF4*-*LIKE4*, and *ID1* (Pearson’s *R* > 0.459, p < 0.005) were also positively correlated. There were many *FRL* genes expressed in the tissue we tested that contain an FRI domain. The floral repressor *FLC* and its activator *FRI* are known to be absent in maize^26^. Interestingly, different *FRL* homologs showed positive correlations with different flowering-pathway genes. For example, *FRL3b* and *EFL3a* (Pearson’s *R* = 0.591, p < 0.001), *FRL3c* and *PHYC1* (Pearson’s *R* = 0.598, p < 0.001), *FRL3d* and *PIE1b* (Pearson’s *R = *0.714, p < 0.001), *FRL4a* and *DLF1* (Pearson’s *R* = 0.623, p < 0.001), and *FRL4c* and *PHYA2* (Pearson’s *R* = 0.534, p < 0.001) showed significant positive correlations. The relationships among these 55 flowering-time genes suggest possible networks in the regulation of flowering time.

## Discussion

To investigate the origin and genetic characteristics of Korean landraces, we used 2,604,272 SNPs from 12 Korean landraces. SNPs in coding sequences obtained from RNA-seq at the early vegetative stage were sufficient to characterize the Korean landraces, even without complete genome sequences. To define the relationships of Korean landrace to other maize lines, we obtained SNPs from HapMap2 data and used PCA and neighbor-joining cluster analysis. From this analysis, we conclude that Korean landraces cluster with a Chinese elite inbred line CAUCHANG72, which is one parent of a hybrid widely cultivated in China, Zhengdan 958^19^. To minimize the distortion among coding sequences and genome sequences of HapMap data caused by differences in the depth of sequencing, quality of the sequences, and sequencing of either mRNA or DNA, we only used regions of the B73 genome where our samples were mapped. HapMap3 also showed that sequence depth variations among samples (5× to 1,000×) were not a problem for pairwise comparison^2^. To our knowledge, this is the first DNA sequence-level analysis of Korean landraces. This analysis could open the way to studying the genetic characteristics and diversity of Korean landraces to enhance their utility as breeding sources.

To maximize genetic diversity among our samples, we collected maize landraces with diverse flowering times. Flowering-time gene profiling showed that there were multiple causes of the variation of flowering time within Korean landraces. The clustered branching of Korean landraces suggests that flowering-time diversification likely occurred after the introduction of the original maize line to Korea. Alternatively, Korean landraces of independent origins could be converging due to repeated crosses. Both the literature and relatively short history of cultivation suggest the former is more likely. The variation in flowering time observed in the Korean landraces was quite small compared to global landraces, which take 2–11 months to mature^27^. These small variations might stem from the relatively short 500 years of cultivation in Korea compared to the 7,500-year history of maize cultivation^28^.

We observed several positive and negative correlations among flowering-time genes by Pearson’s correlation analysis. FRI is known to activate FLC, a floral repressor mainly connected to vernalization. Both FLC and vernalization are absent in maize^26^. *FRL* was cloned by mutant screening in a dominant FRI line of Arabidopsis and identified as a suppressor of FRI-mediated late flowering^29^. Further analysis revealed that FRL is a component of the FRIGIDA complex, which activates transcription of *FLC*^30^. There are 11 *FRL* genes annotated in the B73 maize genome, and the expression of six were detected and analyzed in this study. Questions remain surrounding the divergent expression patterns of the FRL homologues in the absence of active FRI and FLC in maize. None of the genes highly correlated with *FRL* homologues is a known FRI complex component. Our knowledge of *FRL* gene function to date has been limited to the FRI complex^30^. However, the variation in expression patterns of *FRL* genes and their significant correlations with those of flowering-time genes observed in this study suggest that *FRL* genes may have diverged in their function regulating flowering time in maize.

REF6 and ELF6 are Jumonji domain-containing proteins known to function in *FLC* histone modification^31^. They were first identified as highly similar proteins and predicted to be histone demethylases with similar but not identical flowering-time phenotypes^31^. REF6 is a histone H3 lysine 27 demethylase and is involved in the brassinosteroid signaling pathway through protein–protein interactions with BES1^32,33^. Although the exact functions of REF6 and ELF6 in maize were not identified in this study, the significant positive correlation between *REF6* and *ELF6* expression and their slightly different correlations with the expression of other flowering-time genes suggest they play similar but distinct roles in regulating flowering time.

VIP3 is a component of the RNA polymerase-associated factor 1 complex (Paf1c) associated with chromatin remodeling factors^34^. Significant positive correlation among the expression of *D8*, *D9*, and *VIP3* suggests that Paf1c could be involved in the gibberellic acid (GA) pathway in maize. *D8* expression was significantly negatively correlated with expression of *PIE1b* and *FLK2b*. D8 and D9 are DELLA proteins^35^. PIE1 and FLK are also components of the protein complexes that are involved in the autonomous and circadian clock pathways, respectively, suggesting possible connections among the GA, autonomous, and circadian clock pathways, similar to recent reports in rice^36^.

Not all of the genes in this study have known functions, but we can infer potential functions through positive or negative correlations in expression of these genes. Many flowering genes in maize remain functional even without a functional FLC pathway. This enables us to infer the function of unknown genes or suggest interactions between pathways. Detailed analyses of additional developmental stages or tissues would be beneficial for future investigations.

This study showed variations among the Korean landraces, some of which could provide novel genetic resources. Our results also suggest that there has been little germplasm exchange between foreign and Korean maize lines until recently. By analyzing 12 landraces with various flowering times, we showed that the majority of the landraces could be derived from a single origin followed by subsequent genetic and phenotypic variations after maize’s introduction to the Korean peninsula. WGCNA and flowering time-related gene expression profiles suggest that there is not a single causal genotype for early or late flowering of Korean landraces, and some interactions may exist among flowering time-related genes in maize. We only analyzed 12 landraces in this study, and a large-scale investigation of the Korean landraces could provide detailed characterization and breeding resources.

## Supplementary information

Supplementary information.
