# Supplementary material for: A genetic characterization of Korean waxy maize (Zea mays L.) landraces having flowering time variation by RNA sequencing
Source: Sci Rep. 2019 Dec 27;9:20023. doi: 10.1038/s41598-019-56645-y (PMC6934685; doi:10.1038/s41598-019-56645-y)
Supplement: Supplementary file 1 — Supplementary information. [file 41598_2019_56645_MOESM1_ESM.docx]

**A genetic characterization of Korean waxy maize (*Zea mays* L.) landraces having flowering time variation by RNA sequencing**

Gibum Yi^+^*, Hosub Shin^+^, Seung Hwa Yu, Jeong Eun Park, Taegu Kang, and Jin Hoe Huh*

**Supplementary Information**

This Supplementary Information contains 2 Supplementary Tables and 4 Supplementary Figures

**Supplementary Table 1**. Summary of RNA-seq reads for 13 maize lines.

| Sample | reps | Filtered reads (Mbp) | Filtered reads (ea) | Aligned pairs (ea) | Multiple alignments (ea) | Discordant | Rate of concordant pairing (%) |
| --- | --- | --- | --- | --- | --- | --- | --- |
| B73 | 1 | 6,411.8 | 63,483,348 | 27,925,525 | 2,002,662 | 308,125 | 87.00 |
|  | 2 | 6,377.7 | 63,145,724 | 28,134,400 | 2,025,582 | 255,288 | 88.30 |
|  | 3 | 6,538.6 | 64,738,622 | 28,828,005 | 1,956,059 | 239,413 | 88.30 |
| Seocheon Chal | 1 | 3,999.9 | 39,603,014 | 15,869,841 | 1,425,442 | 146,456 | 79.40 |
|  | 2 | 3,866.8 | 38,284,994 | 15,165,823 | 1,356,056 | 149,148 | 78.40 |
|  | 3 | 3,782.7 | 37,452,816 | 14,841,972 | 1,394,332 | 159,896 | 78.40 |
| Pyeongchang Chal-14 | 1 | 3,691.1 | 36,545,704 | 14,253,743 | 982,552 | 236,513 | 76.70 |
|  | 2 | 4,405.8 | 43,621,378 | 17,030,724 | 1,252,459 | 240,326 | 77.00 |
|  | 3 | 3,642.5 | 36,063,862 | 14,112,189 | 1,200,172 | 174,610 | 77.30 |
| Okcheon Chal-1 | 1 | 3,831.9 | 37,940,090 | 14,988,126 | 1,120,663 | 204,092 | 77.90 |
|  | 2 | 3,934.6 | 38,956,592 | 14,856,682 | 1,284,500 | 179,396 | 75.40 |
|  | 3 | 3,872.8 | 38,344,180 | 15,061,319 | 1,131,784 | 216,395 | 77.40 |
| Goseong Chal | 1 | 4,547.0 | 45,019,636 | 17,677,515 | 1,420,524 | 176,123 | 77.80 |
|  | 2 | 4,247.8 | 42,057,448 | 6,591,310 | 1,305,947 | 190,812 | 78.00 |
|  | 3 | 3,837.2 | 37,991,736 | 15,023,349 | 1,221,948 | 155,283 | 78.30 |
| Wonju Chal | 1 | 3,748.7 | 37,115,952 | 14,405,421 | 1,401,378 | 178,618 | 76.70 |
|  | 2 | 3,846.4 | 38,083,116 | 14,755,602 | 1,270,116 | 167,629 | 76.60 |
|  | 3 | 3,912.9 | 38,741,920 | 15,099,192 | 1,325,095 | 168,649 | 77.10 |
| Hoengseong Chal-9 | 1 | 4,190.1 | 41,486,380 | 16,228,522 | 1,193,642 | 225,957 | 77.10 |
|  | 2 | 3,702.4 | 36,657,470 | 14,178,989 | 1,096,661 | 246,359 | 76.00 |
|  | 3 | 4,297.2 | 42,546,374 | 16,513,624 | 1,242,711 | 275,822 | 76.30 |
| Jeongseon Chal-1 | 1 | 4,152.6 | 41,115,316 | 16,042,204 | 1,168,715 | 210,682 | 77.00 |
|  | 2 | 4,857.8 | 48,096,696 | 18,816,695 | 1,306,496 | 295,880 | 77.00 |
|  | 3 | 4,121.8 | 40,809,572 | 15,880,048 | 1,239,337 | 225,028 | 76.70 |
| Inje Chal-1 | 1 | 4,555.8 | 45,106,774 | 17,467,428 | 1,394,026 | 264,435 | 76.30 |
|  | 2 | 3,947.5 | 39,084,058 | 15,175,619 | 1,280,475 | 217,320 | 76.50 |
|  | 3 | 4,469.8 | 44,255,932 | 17,225,419 | 1,272,002 | 242,007 | 76.80 |
| Cheongyang Chal | 1 | 4,552.7 | 45,076,500 | 17,686,464 | 1,463,795 | 184,572 | 77.70 |
|  | 2 | 4,156.2 | 41,150,842 | 16,257,076 | 1,281,075 | 177,461 | 78.10 |
|  | 3 | 4,002.2 | 39,625,648 | 15,628,223 | 1,243,176 | 158,884 | 78.10 |
| Hoengseong Chal-3 | 1 | 3,796.5 | 37,588,820 | 14,419,529 | 995,249 | 193,453 | 75.70 |
|  | 2 | 3,826.1 | 37,881,872 | 14,884,908 | 1,046,991 | 182,001 | 77.60 |
|  | 3 | 3,966.9 | 39,276,640 | 15,355,302 | 1,210,018 | 163,373 | 77.40 |
| Dangjin Chal | 1 | 4,739.8 | 46,929,154 | 18,153,342 | 1,181,626 | 303,166 | 76.10 |
|  | 2 | 3,724.5 | 36,876,614 | 14,258,698 | 959,165 | 226,921 | 76.10 |
|  | 3 | 4,103.6 | 40,630,088 | 15,649,178 | 1,146,264 | 265,534 | 75.70 |
| Misang Chal-8 | 1 | 4,576.8 | 45,314,812 | 17,777,696 | 1,546,654 | 202,935 | 77.60 |
|  | 2 | 4,425.9 | 43,820,570 | 17,071,833 | 1,403,948 | 222,958 | 76.90 |
|  | 3 | 3,830.5 | 37,926,144 | 14,738,744 | 1,266,726 | 188,937 | 76.70 |

**Supplementary Table 2**. List of flowering time-related genes used in this study

| Gene ID | Pathway | Description^*^ | Gene name^+^ | Module^#^ |
| --- | --- | --- | --- | --- |
| Zm00001d004875 | circadian | APRR9 | *ZmPRR59* | yellow |
| Zm00001d005107 | autonomous | FRIGIDA-like 3 | *FRL3a* | brown |
| Zm00001d006752 | autonomous | FRIGIDA-like 3 | *FRL3b* | brown |
| Zm00001d007240 | circadian | ZmPRR37 | *ZmPRR37* | red |
| Zm00001d007835 | autonomous | FRIGIDA-like 4a | *FRL4a* | blue |
| Zm00001d008826 | circadian | Gigantea1 | *GI1* | yellow |
| Zm00001d010987 | integrator | ZmRAP2.7 | *ZmRAP2.7* | light yellow |
| Zm00001d011876 | photoperiod | ZmHy2 | *ZmHY2* | yellow |
| Zm00001d013262 | photoperiod | PhyC2 | *PHYC2* | green |
| Zm00001d013402 | photoperiod | PhyA2 | *PHYA2* | red |
| Zm00001d013465 | GA | Dwarf plant 9 | *D9* | red |
| Zm00001d013597 | autonomous | CDC73/PHP | *CDC73* | blue |
| Zm00001d013963 | circadian | FLK homology 2a | *FLK 2a* | black |
| Zm00001d017660 | autonomous | PHOTOPERIOD-INDEPENDENT EARLY FLOWERING 1 | *PIE1a* | tan |
| Zm00001d018142 |  | ZMM26 | *ZMM26* | pink |
| Zm00001d018669 | circadian | CK2 protein kinase alpha 4 | *CK2a4* | yellow |
| Zm00001d018911 | autonomous | FRIGIDA-like 4a | *FRL4b* | black |
| Zm00001d019039 | autonomous | SHL1 | *SHL1* | green |
| Zm00001d019890 | autonomous | FRIGIDA-like 3 | *FRL3c* | cyan |
| Zm00001d020036 | floral gene | Embryonic flower 2a | *EMF2a* | red |
| Zm00001d020364 | circadian | Early flowering 4a | *EFL4a* | black |
| Zm00001d021188 | autonomous | FPA | *FPA* | black |
| Zm00001d022016 | autonomous | FRIGIDA-like 3 | *FRL3d* | blue |
| Zm00001d022590 | circadian | ZmPRR37 | *ZmPRR37* | red |
| Zm00001d022613 | integrator | Delayed flowering1 | *DLF1* | turquoise |
| Zm00001d023788 | autonomous | REF6 | *REF6* | light green |
| Zm00001d024546 | circadian | CCA2 | *CCA2* | magenta |
| Zm00001d028173 | autonomous | FRIGIDA-like 1 | *FRL1* | blue |
| Zm00001d028905 | photoperiod | PhyB1 | *PHYB1* | black |
| Zm00001d030968 | circadian | FLK homology 2b | *FLK2b* | midnight blue |
| Zm00001d032922 | autonomous | ID1 | *ID1* | black |
| Zm00001d033680 | GA | Dwarf plant 8 | *D8* | black |
| Zm00001d033799 | photoperiod | PhyA1 | *PHYA1* | salmon |
| Zm00001d034036 | floral gene | Embryonic flower 2b | *EMF2b* | blue |
| Zm00001d034038 | photoperiod | PhyC1 | *PHYC1* | blue |
| Zm00001d034858 | autonomous | FRIGIDA | *FRI* | midnight blue |
| Zm00001d037565 | GA | Gibberellin 2-oxidase1 | *GA2OX1* | tan |
| Zm00001d039156 | circadian | Early flowering 3a | *EFL3a* | blue |
| Zm00001d039589 | circadian | Gigantea2 | *GI2* | purple |
| Zm00001d039594 | autonomous | FRIGIDA-like 4a | *FRL4c* | brown |
| Zm00001d042091 | autonomous | FY | *FY* | green |
| Zm00001d042212 | autonomous | Luminidependens protein1 | *LDP1* | light cyan |
| Zm00001d044232 | circadian | Early Flowering 3b | *EFL3b* | green |
| Zm00001d045735 | photoperiod | CONSTANS-LIKE 1 | *CONZ1* | purple |
| Zm00001d045944 | photoperiod | Cryptochrome2 | *CRY2* | black |
| Zm00001d047269 | circadian | Early flowering 4b | *EFL4b* | brown |
| Zm00001d047632 | photoperiod | PhyB2 | *PHYB2* | tan |
| Zm00001d047761 | circadian | APRR3 | *APRR3* | red |
| Zm00001d048404 | autonomous | ELF6 | *ELF6* | yellow |
| Zm00001d048474 | integrator | ZMM5 | *ZmMADS1* | pink |
| Zm00001d048691 | autonomous | VIP3 | *VIP3* | yellow |
| Zm00001d048801 | circadian | ELF4-like 4 | *ELF4-LIKE4* | black |
| Zm00001d049543 | circadian | Circadian clock associated1 | *CCA1* | black |
| Zm00001d051507 | autonomous | PHOTOPERIOD-INDEPENDENT EARLY FLOWERING 1 | *PIE1b* | brown |
| Zm00001d052180 |  | tunicate1 | *ZMM19* | yellow |

***** Modified from the descriptions of gramene.org

+ When the genes had the same description, a suffix was added, e.g. a, b, c, etc.

# Modules were defined by weighted gene co-expression network analysis (WGCNA)


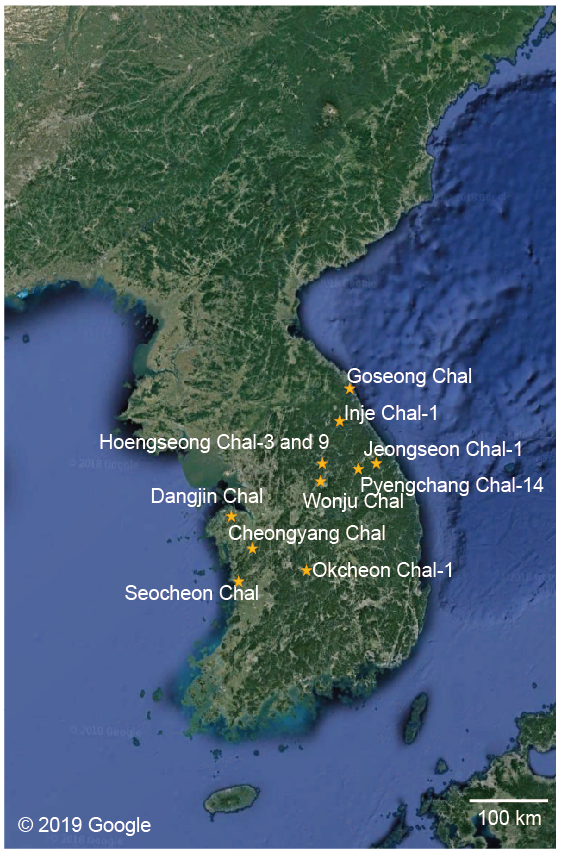


**Supplementary Figure S1**. Geographical sampling locations of the Korean landraces. Hoengseong Chal-3 and Hoengseong Chal-9 originated from the same location. Misang Chal-8 has an unknown geographic origin. The locations are positioned by their longitude and latitude on a satellite map of the Korean peninsula form Google Earth (Map data © https://earth.google.com/web/@38.07058385,125.96816036,389.90479495a,1977957.63301313d,35y,359.99999879h,0t,0r). The map was edited with Adobe Illustrator CS6 (version 16.0.0).


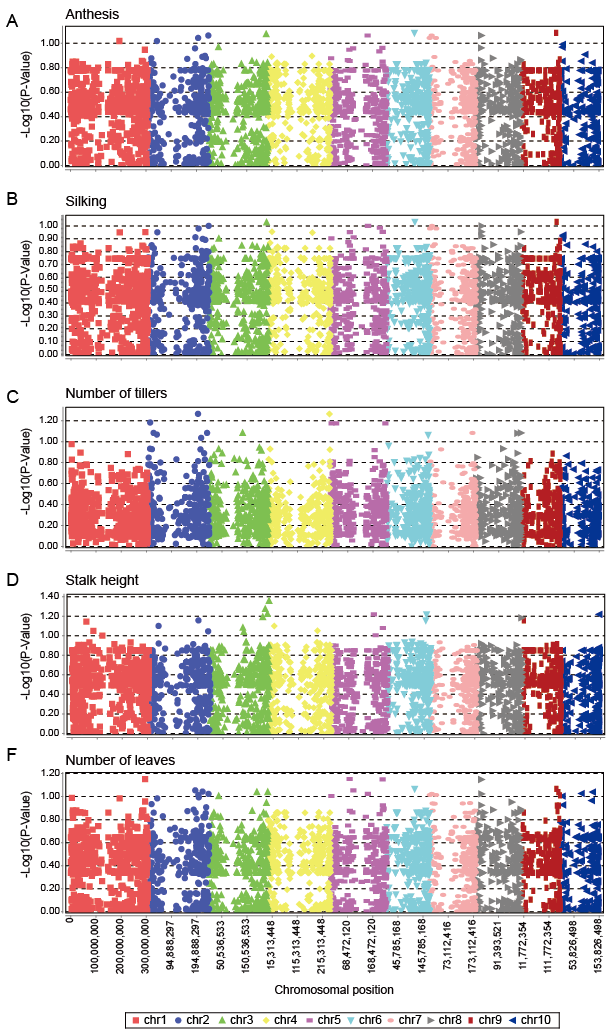


**Supplementary Figure S2**. Genome-wide association study of five phenotypes and SNP genotypes of the 13 lines. Plots are showing the significance of the association between SNP genotypes and quantitative phenotypes across the genome which colored and shaped differently according to their chromosomal positions. Phenotypes are indicated at the top left side of the plots: (A) Anthesis, (B) Silking, (C) Number of tillers, (D) Stalk height, and (E) Number of leaves.


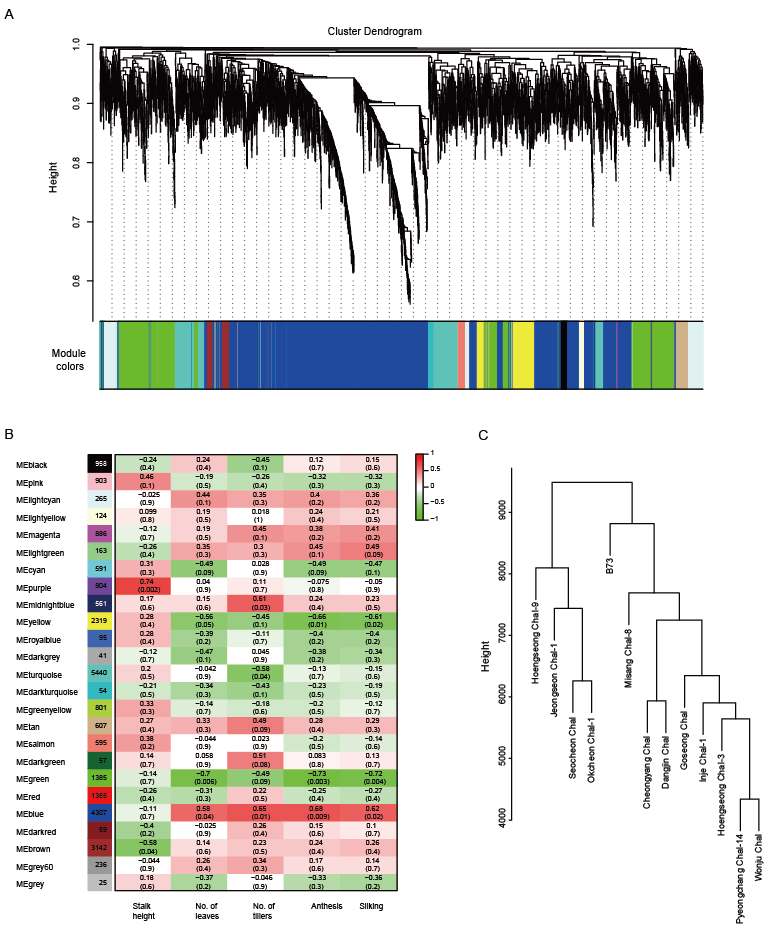


**Supplementary Figure S3**. Weighted gene co-expression network analysis of Korean maize landraces. (A) Modules were defined from network construction and the hierarchical clustering. (B) Color scaled correlations between the twenty-four modules and phenotypes. Corresponding *p*-values are in parenthesis. The numbers in module color on the left column indicate the number of genes belong to each module. (C) Sample clustering based on their transcriptome profiles which was used for the weighted gene co-expression network analysis.


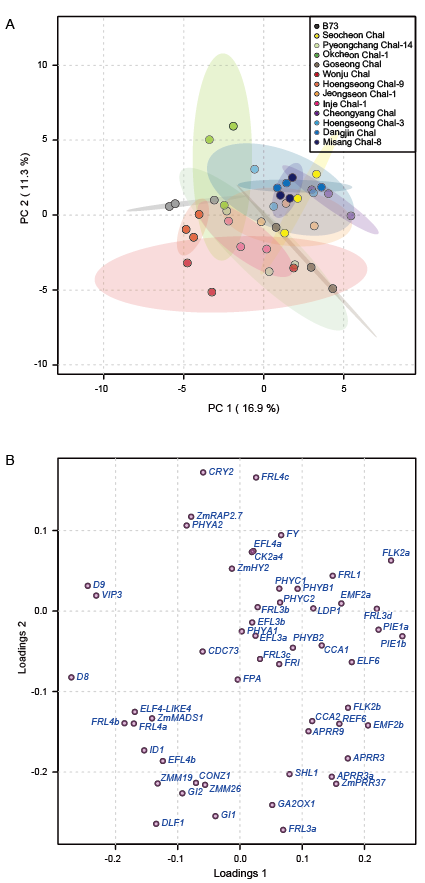


**Supplementary Figure S4**. Principal component analysis based on the expression of 55 flowering-time genes. FPKM values of the genes were auto scaled and used for PCA. (A) Score plot of three replicates of 12 Korean landraces and B73. Colored areas show 95% confidence intervals. (B) Loading plot of 55 flowering-time genes on PC1 and PC2.
